# Propofol Suppresses Ferroptosis via Modulating eNOS/NO Signaling Pathway to Improve Traumatic Brain Injury

**DOI:** 10.1002/brb3.70187

**Published:** 2024-12-26

**Authors:** Zi‐Lei Zheng, Xu‐Peng Wang, Yu‐Fei Hu, Wen‐Guang Li, Qi Zhou, Fang Xu, Qiu‐Jun Wang

**Affiliations:** ^1^ Department of Anesthesiology The Third Hospital of Hebei Medical University Shijiazhuang China; ^2^ Department of Anesthesiology The No.4 Hospital of Zhangjiakou Zhangjiakou China; ^3^ Graduated School Hebei Medical University Shijiazhuang China

**Keywords:** eNOS, ferroptosis, nitric oxide, propofol, traumatic brain injury

## Abstract

**Purpose:**

This study aims to explore the neuroprotective effect of propofol in improving traumatic brain injury (TBI) by inhibiting ferroptosis through the modulation of the endothelial nitric oxide (NO) synthase (eNOS)/NO signaling pathway.

**Methods:**

The GSE173975 dataset was used to analyze the differentially expressed genes between TBI and sham surgery control groups in the short and long term. A TBI model was established in 2‐month‐old male SPF C57BL/6 mice by impact exposure of the exposed dura mater. After the establishment of the TBI model, propofol (30 mg/kg) or saline was administered via intraperitoneal injection for intervention. Nissl staining and Perls staining were employed to assess neuronal function and iron deposition, respectively. Western blot technology was employed to detect the expression of proteins related to ferroptosis. Immunofluorescence staining of astrocytes and microglia was utilized to assess the neuroinflammatory response induced by TBI. The Morris water maze (MWM) and novel object recognition (NOR) tests were employed to assess cognitive dysfunction induced by TBI.

**Findings:**

Bioinformatics analysis revealed aberrant gene expression associated with iron transport, neuronal death, and inflammatory response in the initial stages of TBI. Long‐term abnormalities were predominantly linked to genes involved in inflammatory response. Perls staining and protein expression analysis confirmed the occurrence of iron deposition and ferroptosis following TBI. Propofol treatment significantly reduced iron deposition and ferroptosis induced by TBI. Nissl staining demonstrated enhanced neuronal function, while TUNEL staining indicated reduced neuronal apoptosis. Immunofluorescence analysis demonstrated that propofol significantly reduced the proliferation of astrocytes and activation of microglia induced by TBI in the long term. The results of MWM and NOR tests indicated that propofol significantly improved the long‐term cognitive dysfunction induced by TBI. Propofol exerts neuroprotective effects by increasing the expression of eNOS protein and the content of NO. The neuroprotective effects of propofol can be reversed by the eNOS inhibitor L‐NAME.

**Conclusion:**

Propofol significantly improves the prognosis of TBI by inhibiting ferroptosis through the modulation of the eNOS/NO signaling pathway. The study results provide a scientific basis for the clinical use of propofol as a neuroprotective agent and offer a new direction for the development of new treatment strategies for TBI.

## Graphical abstract

This study established a TBI model using a high‐altitude free‐fall method and found ferroptosis plays a significant role in the acute phase of TBI, with inflammation present throughout. Propofol alleviates inflammation and improves cognitive deficits by inhibiting ferroptosis via the eNOS/NO signaling pathway.

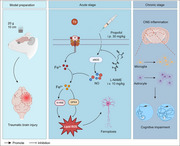

## 1 Introduction

Traumatic brain injury (TBI) is a pervasive and grave public health issue of global significance due to its high prevalence and associated risks (Dewan et al. 2018). TBI can result from a variety of situations, such as head injuries sustained in sports, falls, auto accidents, and traffic incidents (Capizzi, Woo, and Verduzco‐Gutierrez 2020; Zetterberg et al. 2019). According to the Lancet Neurology Commission (2022), TBI affects more than 55 million people globally and is a major cause of morbidity and mortality (Maas et al. 2022). TBI can induce long‐term physical and cognitive complications that can seriously affect the quality of life of patients (Kowalski et al. 2021; McCrea et al. 2021). Several studies have indicated that the economic burden associated with TBI exceeds $400 billion per year. Consequently, it is of the utmost importance to reduce the prevalence of TBI and to improve treatment and rehabilitation strategies (Dewan et al. 2016; Peeters et al. 2015). By understanding the pathogenesis of TBI, it will be possible to provide a conceptual framework for medical care. This will not only improve the prognosis of TBI but will also be of significant benefit in curbing the global healthcare expenditures associated with TBI.

The pathogenesis of TBI is complex. TBI causes not only primary tissue damage but also secondary damage, including neuroinflammation, oxidative stress, and excitotoxicity (Bagri, Kumar, and Deshmukh 2021). TBI leads to neuronal damage and death not only at the site of the lesion but also in neighboring tissues that are not directly affected by the mechanical force (Batsaikhan et al. 2019; J. Wang et al. 2021). Ferroptosis is a novel type of programmed cell death that differs from apoptosis, pyroptosis, and autophagy. It is characterized by an excessive concentration of iron ions and oxidative stress (Y. Miao et al. 2022; Y. Wang, Zhang, Bi, et al. 2022). Iron ions play a key role in normal cellular metabolism; however, their excessive presence triggers oxidative stress, which disrupts cellular oxidative homeostasis and ultimately leads to damage to lipids, proteins, nucleic acids, and other biomolecules. Numerous illnesses, including neoplasms, cardiovascular conditions, and neurological disorders, have been linked to ferroptosis‐induced cell death (Badgley et al. 2020; Ryan et al. 2023). A differentially expressed gene (DEG) module that was upregulated during the acute phase (1 day) of TBI was associated with cell death and astrocytosis. This was measured by high‐throughput sequencing analysis by Catta‐Preta et al. (2021). According to recent research, ferritin deposition is thought to be a critical component in the damage cascade in cases of cranial brain injury. Following cranial injury, cells experience acute hypoxia and oxidative stress, leading to an abnormal accumulation of intracellular ferric ions. These ions interact with free radicals and other molecules, leading to oxidative stress and intracellular molecular damage, ultimately resulting in cellular ferroptosis (D. Wang, Zhang, Ge, et al. 2022). The study by J. Chen, Wang, et al. (2024) demonstrates that Netrin‐1 can mitigate early brain injury induced by subarachnoid hemorrhage by enhancing the peroxisome proliferator‐activated receptor γ (PPARγ), a mechanism associated with PPARγ's regulation of lipid metabolism to reduce ferroptosis. Therefore, identifying targeted inhibition of oxidative stress and lipid peroxidation‐induced ferroptosis may be crucial for improving the prognosis of TBI.

Propofol is a widely used general anesthetic worldwide. Recent scientific research has revealed the potential role of propofol in neuroprotection, opening new avenues for its clinical application (Kaur, Flores Gutierrez, and Nistri 2016; Yu et al. 2020). In particular, studies of neurological disorders such as stroke have demonstrated that propofol is able to attenuate apoptosis, thereby reducing ischemia‐induced cell death and, to some extent, brain damage (Kotani et al. 2008; B. Sun et al. 2018). Furthermore, propofol has been demonstrated to modulate endothelial nitric oxide (NO) synthase (eNOS) expression and attenuate myocardial ischemia‐reperfusion injury (Zuurbier et al. 2014). The study by Fan et al. (2023) demonstrates that propofol has been proven to inhibit ferroptosis in a mouse model of cerebral ischemia‐reperfusion via the Nrf2/Gpx4 signaling pathway, suggesting that propofol may have a protective effect against cerebral ischemia‐reperfusion injury. While the neuroprotective effects of isoproterenol in controlling stroke and myocardial ischemia‐reperfusion injury have been recognized to a certain extent, its effect on eNOS expression in a TBI model, and the relationship between this effect and ferroptosis, including the underlying molecular signaling pathways, remains an unexplored area. The objective of this study was to ascertain whether isoproterenol could mitigate TBI‐induced iron toxicity by modulating eNOS expression and to delineate the potential signaling pathways involved. It is our hope that this study will provide new insights and strategies for the treatment of TBI, particularly with regard to reducing or preventing TBI‐induced ferritin deposition and neurological damage.

## 2 Materials and Methods

### 2.1 Animals

This experiment utilized male SPF C57BL/6 mice aged 2 months, weighing between 22 and 26 g. Liaoning Immortality Biological Technology Co. Ltd. provided the mice (license: SCXK (Liao) 2023‐KY‐039). They were housed in clean cages with regular bedding changes. The housing chamber maintained a temperature between 22°C and 28°C, relative humidity between 40% and 60%, and ran on a 12‐h light‐and‐dark cycle. Throughout the study, mice had unlimited access to food. The National Institutes of Health's Guide for the Treatment and Utilization of Laboratory Animals was followed in all animal experiments. The Hebei Medical University Animal Review Board's Third Hospital provided ethical approval for all mouse‐related research (Z2022‐034‐1).

### 2.2 Microarray Data Sets

To obtain the GSE173975 dataset, the Gene‐Expression Omnibus (GEO, https://www.ncbi.nlm.nih.gov/geo) database was utilized. RNA‐seq data were selected from the GSE173975 dataset for further analysis. The data were obtained from the GPL22396 (Illumina HiSeq 4000) platform and contained mRNA information for hippocampal samples from TBI and sham‐operated control rats at 1 and 14 days after brain injury. After downloading the series matrix files, the data were subjected to quantile normalization using R (version R 4.4.1) software. To find DEGs in the TBI and sham‐operated control rats' data, we used R's limma package. A *p* < 0.05 and |log_2_ (Fold Change, FC)| > 1 were the screening criteria for DEGs.

### 2.3 TBI Model

Mice were placed in an anesthesia chamber that had been prefilled with 8% sevoflurane (21102331; Hearem, Shanghai, China). Once the righting reflex had disappeared, the mice were positioned in the prone position using a stereotaxy instrument (ZS‐FD/S; Zhongshi Science and Technology, Beijing, China), and anesthesia was maintained with 3% sevoflurane. Before the procedure began, the hair on the mice's heads was shaved off, and the area was disinfected using iodine and alcohol. A longitudinal incision of approximately 1.5 cm was made along the midline of the head, this incision allowed for the separation of the soft tissue and periosteum while exposing the skull. Using a cranial drill, a 3.5 mm spherical bone window was made on the right side of the skull's midline, between the coronal and sagittal sutures, without causing any damage to the dura mater. Next, a 20 g metal weight was dropped vertically from a height of 10 cm onto a 3.0 mm diameter and 2.5 mm thick cylinder placed on the dura, producing an impact force of 200 g/cm. This impact caused a contusion and laceration injury to the right parietal cortex, covering an area of 3.0 mm × 3.0 mm. After applying bone glue to seal the bone window, the scalp incision was sutured. Once the mice were conscious again, they were free to consume. The mice in the control group did not experience the impact despite undergoing a similar surgical operation. Propofol intervention was conducted 30 min after the preparation of the model by intraperitoneal injection of 0.05% propofol at a dose of 30 mg/kg (23042412; Sichuan Guorui Pharmaceutical Co. Ltd., Leshan, China). The control group received an equivalent volume of saline. In subsequent experiments, before the application of propofol, eNOS protein inhibitor L‐NAME (S2877, Selleck, USA) was administered intraperitoneally at a dose of 30 mg/kg, or an equivalent volume of solvent was injected as a control.

### 2.4 Nissl Staining

Three days after TBI, six mice in each group were chosen at random and euthanized under deep sevoflurane anesthesia, followed by the extraction of their brain tissues for histological sectioning (*n* = 6). In brief, the brain tissue was preserved with a 4% paraformaldehyde buffer for an entire night. Subsequently, routine alcohol dehydration, paraffin embedding, and sectioning were performed on the cortical lesion area at a thickness of 4 µm, followed by Nissl staining. The sections were submerged in Nissl staining solution (C0117; Beyotime, Shanghai, China) for 15 min following gradient alcohol dewaxing, washed twice with double‐distilled water (ddH_2_O), dehydrated with gradient ethanol, clarified with xylene, and finally mounted with neutral balsam. Four fields from each section were selected for cell counting, and the area of typical Nissl‐stained positive cells was measured using ImageJ software.

### 2.5 Perls Staining

Take the prepared brain slices for Perls staining (*n* = 6). Perls staining was utilized to detect iron ion deposition in the surrounding brain tissue. Paraffin sections were deparaffinized, washed, and then placed in a solution configured with equal volumes Perls A dye and B dye (G1029; Servicebio, Wuhan, China) for 30 min and spent 5 min washing with ddH_2_O. The slices were then dyed for 30 s using Perls C dye and then washed with ddH_2_O for another 30 s. Finally, the slices were fixed by routine dehydration and transparent, and neutral gum sealing. Four fields were selected from each section for counting of Perls‐stained positive cells, performed using ImageJ software.

### 2.6 Western Blot

On the third day after TBI, six mice were chosen randomly from every group (*n* = 6). After deep anesthesia with sevoflurane, the chest cavity was opened to expose the heart, and ice‐cold physiological saline was perfused through the aorta until the liquid from the right auricle became clear. The brain of the injured side of the mouse was then dissected. The total protein of the brain tissue was separated and measured using a BCA Assay Kit (P0011; Beyotime, Shanghai, China). Heating proteins for 5 min at 95°C denatured them. For electrophoresis, 30 mg of protein were added to the wells of an SDS‐PAGE gel. Following that, the proteins from the SDS‐PAGE gel were placed onto a 0.45 µm PVDF membrane (Millipore Corporation, Burlington, MA, USA). After blocking the PVDF membrane for 10 min with QuickBlock solution (P0252; Beyotime, Shanghai, China), the primary antibodies were incubated with the membrane for an additional night at 4°C. The primary antibodies utilized in this study include: a rabbit anti‐Bak polyclonal antibody at a dilution of 1:1000 (AB016; Beyotime, Shanghai, China), a rabbit anti‐Bcl‐2 polyclonal antibody at a dilution of 1:1000 (AB112; Beyotime, Shanghai, China), a rabbit anti‐Gpx4 polyclonal antibody at a dilution of 1:1000 (WL05406; Wangleibio, Shenyang, China), a rabbit anti‐4‐HNE polyclonal antibody at a dilution of 1:1000 (MA5‐27570; Thermo Fisher Scientific, MA, USA), a rabbit anti‐eNOS polyclonal antibody at a dilution of 1:1000 (WL01789; Wanglei, Shenyang, China), and a rabbit anti‐α‐tubulin polyclonal antibody at a dilution of 1:1000 (AF0001; Beyotime, Shanghai, China). Following three 5‐min TBST washes, goat anti‐rabbit IgG (1:1500, GB23303; Servicebio, Beijing, China) tagged with peroxidase was incubated for 1 h at 25°C on the PVDF membrane. An ECL Chemiluminescence Detection Kit (180‐5001; Tanon, Shanghai, China) was used for identifying the protein bands and captured with a chemiluminescence detector (MiniChemi610; Sinsage, Beijing, China). Quantitative analysis was performed using ImageJ software.

### 2.7 Immunofluorescence

After the behavioral testing was finished, six mice at random from each group were put to death by decapitation and brain removal while under deep anesthesia with 8% sevoflurane (*n* = 6). After that, the brains were preserved for 12 h in 4% paraformaldehyde. Afterward the brain tissues were transferred to a 30% sucrose solution until they sank. Frozen sections with a thickness of 40 µm were then made. The sections were blocked with 10% goat serum after being treated with 0.3% Triton X‐100 to permeabilize the membranes. Three PBS rinses lasting 5 min each were then performed. Afterward, the sections were incubated at 4°C overnight. The primary antibodies used in this study were rabbit polyclonal Iba1 antibody (1:250, GB153502‐100; Servicebio, Wuhan, China) and rabbit polyclonal GFAP antibody (1:250, AF1177; Beyotime, Shanghai, China). The sections were incubated with goat anti‐rabbit Cy3‐conjugated secondary antibody (1:1000, A0516; Beyotime, Shanghai, China) for 1 h at 25°C in the dark following three washes with PBS lasting 5 min apiece. The slices were mounted using 4′,6‐diamidino‐2‐phenylindole dihydrochloride (DAPI)‐containing anti‐fluorescence quenching mounting solution (P0131; Beyotime, Shanghai, China) following three PBS washes. Finally, the sections were imaged under a confocal microscope (CSIM100, Beijing, China), and the images were quantitatively analyzed using ImageJ software.

### 2.8 TUNEL Staining

Prepared frozen slices were washed with PBS three times for 5 min each. The samples were subsequently incubated for 5 min at 25°C in a PBS solution containing 0.5% Triton X‐100. After three rinses with PBS, rabbit polyclonal NeuN antibody (1:250, GB11138‐100; Servicebio, Beijing, China) was applied and incubated at 4°C for 12 h. Consequently, after three additional washes with PBS and a 60‐min incubation with 50 µL TUNEL assay reagent at 37°C in the dark to detect DNA fragmentation, the sections were rinsed again with PBS. The sections were then treated with a Cy3‐conjugated goat anti‐rabbit secondary antibody (1:1000 dilution, A0516; Beyotime, Shanghai, China) for 1 h at 25°C. Finally, following three rinses with PBS, the slices were mounted with DAPI, imaged using a confocal microscope, and quantitatively analyzed with ImageJ software.

### 2.9 Morris Water Maze

The water maze is constructed of a 1.2‐m‐wide circular pool filled with water up to a height of 40 cm. The water temperature is set at 25 ± 1.0°C. White dye is added to the water to make it opaque, facilitating the precise tracking of the mouse's movement trajectory. The pool is separated into four sections (NE, NW, SE, and SW), with a platform located 1 cm below the water's surface. For the first 4 days, the hidden platform test is conducted: a quadrant is randomly selected from the four for each trial, and the mouse is placed in the water with its back to the center of the pool. Each quadrant is used once during the day's trials, with the order of quadrants randomized daily. Once the mouse finds the platform, it is allowed to rest on it for 30 s. Once the mouse misses to locate the platform within 90 s, it is placed on it and given a 30‐s break. Each mouse goes through four trials every day for 4 days in a row, and the escape latency is recorded using tracking software (Zhongshi Science and Technology, Beijing, China). On the fifth day, a spatial probe test is performed: the platform is removed, and each mouse is placed in the pool to be tracked for 90 s. The number of times the mouse passes the previous position is recorded, as is the duration spent in the quadrant where the platform was placed.

### 2.10 Novel Object Recognition

Following the MWM test, a novel object recognition (NOR) experiment was conducted. Initially, a familiarization phase was undertaken: Two comparable items (A and B) were arranged in symmetrical places in the arena. The mouse was positioned afterward in the center of the two items, allowing consumers 5 min of unfettered exploration. After a 2 h rest period, one recognized object (B) was replaced with a new thing (C). The mouse was again put in the center of the two items for an additional 5 min of unrestricted exploration. A computer tracking system recorded the exploration behavior. Memory ability was assessed by calculating the mouse's discrimination index during the test phase (Discrimination Index = Time exploring the novel item divided by (Time investigating the novel item + Time investigating the familiar item)).

### 2.11 Measurement of NO Concentration in Brain Tissue

Extract 200 mg (*n* = 6) from the obtained brain tissue and thoroughly homogenize. Then, centrifuge at 4°C and 2500 × *g* for 10 min, and collect the supernatant. To perform the process, adhere to the producer's guidelines (A013‐1‐1; Nanjing Jiancheng Bioengineering Institute, Nanjing, China). Using a spectrophotometer, measure the absorbance at 550 nm and calculate the required values.

### 2.12 Glutathione Assay

The GSH Detection Kit (S0053; Beyotime, Shanghai, China) is used to measure the content of glutathione (GSH) in brain tissue. The specific operation method should be carried out according to the manufacturer's instructions.

### 2.13 Malondialdehyde Assay

The MDA Detection Kit (S0131S; Beyotime, Shanghai, China) is used to measure the content of malondialdehyde (MDA) in brain tissue. The specific operation method should be carried out according to the manufacturer's instructions.

### 2.14 Reactive Oxygen Species Assay

To detect the levels of reactive oxygen species (ROS) in mouse brain tissue after TBI, brain tissue samples were collected and prepared into a suspension of single cells 3 days after TBI. Then, the ROS detection kit was used at 37°C with a 30‐min incubation period according to the manufacturer's guidelines (S0035S; Beyotime, Shanghai, China). The levels of ROS in the samples were determined using flow cytometry.

### 2.15 Extraction of RNA and Quantitative Real‑Time PCR

Total RNA was extracted from brain tissue using the Trizol method. Following the manufacturer's instructions, cDNA was synthesized from 500 ng of total RNA using the cDNA Reverse Transcription Kit (D7168M). Q‐PCR was performed using Fast SYBR Green PCR Master Mix (D7260). RT‐PCR was conducted on the QuantStudio 5 Real‐Time PCR System. The expression of FTH1 was normalized to that of GAPDH. Primer information can be found in Table S1.

### 2.16 Statistical Analysis

Data analysis was carried out using GraphPad Prism 9.4.1 (GraphPad Software Inc., San Diego, CA, USA). The Shapiro–Wilk test was used to determine the normality of the data, and the results are shown as mean ± standard deviation (SD). Student *t* tests were employed to compare data between the two groups. The Levene test was utilized to assess the homogeneity of variances in the four data sets, and logarithmic transformation was applied to address any heteroscedasticity. One‐way analysis of variance (ANOVA) tests were performed to analyze the data among the four groups, followed by Tukey tests for post hoc comparisons. The variations in escape latency of mice at different time periods in the MWM are analyzed using two‐way repeated measures ANOVA. Statistical significance was determined at *p *< 0.05.

## 3 Results

### 3.1 Ferroptosis Is a Significant Contributor to Early Pathology of TBI

**FIGURE 1:**
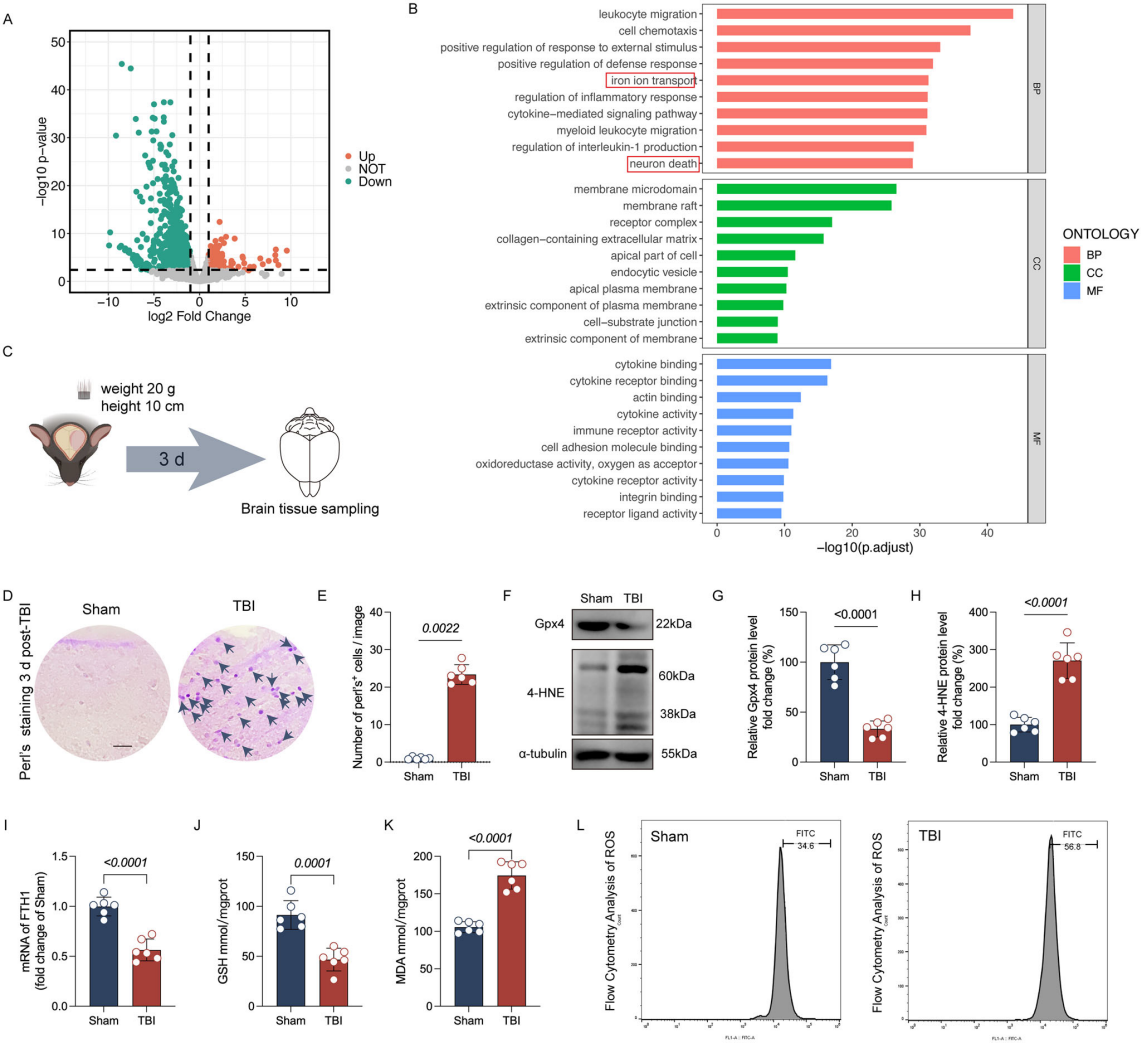
Ferroptosis as a significant contributor to TBI Pathology. (A) One‐day post TBI, a volcano plot was utilized to illustrate the differentially expressed genes between the sham and TBI groups, with the criteria of |log_2_ (fold change, FC)| ≥ 1 and a *p* < 0.05 to distinguish upregulated and downregulated genes. (B) Bar charts were used to present the results of the gene ontology (GO) enrichment analysis. (C) TBI model was prepared by impacting the exposed dura mater after craniotomy with a 20 g weight dropped from a height of 10 cm, and brain tissue sampling was conducted 3 days post‐model preparation. (D) Representative images of Perls staining from both groups of mice. (E) Comparison of the number of positively stained cells in Perls staining between the two groups of mice (*n* = 6 per group). (F) Representative immunoblots of Gpx4 and 4‐HNE proteins from both groups of mice are shown. (G, H). Comparison of the expression levels of Gpx4 and 4‐HNE proteins between the two groups of mice (*n* = 6 per group). (I) Comparison of the expression levels of FTH1 mRNA between the two groups of mice (*n* = 6 per group). (J, K) Comparison of the levels of GSH and MDA between the two groups of mice (*n* = 6 per group). (L) Representative images of flow cytometry analysis of ROS (*n* = 6 per group). Data are represented as mean ± standard deviation, and statistical analysis was performed using Student's *t*‐test, with *p* values indicated directly on the bar charts.

We analyzed the DEGs between TBI and the sham group (1 day) in the GSE173975 dataset, identifying 266 upregulated and 849 downregulated genes in the acute stage of TBI (Figure 1A). GO enrichment analysis revealed that the acutely DEG modules were closely related to “neuronal death,” “iron ion transport,” and immune response (Figure 1B). These findings suggest that iron transport disruption may be an important driver in the progression of TBI. As iron deposition is a key characteristic of ferroptosis, we collected brain tissue samples at 3 days post‐TBI and confirmed iron deposition using Perls staining (Figure 1C–E). To further establish the role of ferroptosis in the pathological process of TBI, we measured the expression levels of the ferroptosis marker proteins Gpx4 and 4‐HNE. The results showed decreased Gpx4 expression and increased 4‐HNE expression in the TBI group compared to the sham group (Figure 1F–H). To further confirm ferroptosis characterized by lipid peroxidation, we detected the levels of GSH, MDA, and ROS and also examined the mRNA levels of the iron storage protein FTH1. The results showed that TBI caused a decrease in FTH1 mRNA expression, a reduction in GSH levels, and an increase in MDA and ROS levels (Figure 1I–L). These results indicate that ferroptosis is a significant contributor to early‐stage TBI injury.

### 3.2 Propofol Can Inhibit Ferroptosis and Exert Neuroprotective Effects

**FIGURE 2:**
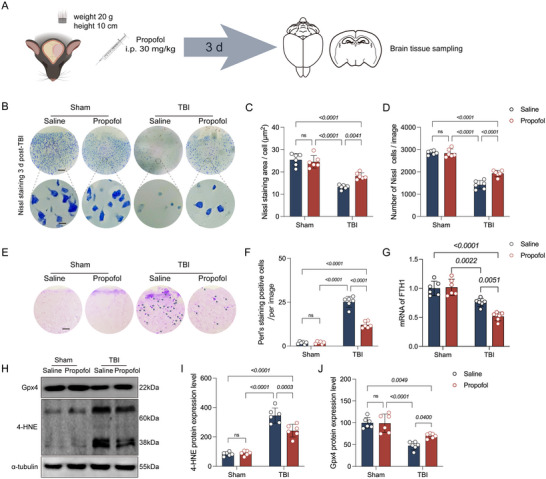
Propofol exerts neuroprotection by inhibiting ferroptosis. (A) Schematic of the experimental procedure. Following the establishment of the TBI model, intervention was conducted via intraperitoneal injection of propofol (dose: 30 mg/kg), with brain tissue samples collected 3 days post‐intervention. (B) Representative Nissl staining images for the four groups of mice, with scale bars = 50 or 10 µm. (C, D) Comparative analysis of Nissl body count and positive area per field of view for the four groups of mice (*n* = 6 per group). (E) Representative images of Perls staining for the four groups of mice. (F) Comparative analysis of the number of positively stained cells in Perls staining for the four groups of mice (*n* = 6 per group). (G, H) Representative immunoblots of Gpx4 and 4‐HNE proteins from the four groups of mice. (G) Comparison of the expression levels of FTH1 mRNA between the four groups of mice (*n* = 6 per group). (I, J) Comparative analysis of the expression levels of Gpx4 and 4‐HNE proteins for the four groups of mice (*n* = 6 per group). Data are presented as mean ± standard deviation, and statistical analysis was conducted using ANOVA, with *p* values directly indicated on the bar charts.

Having established that ferroptosis is a major contributor to TBI, characterized by lipid peroxidation, and given the substantial evidence that propofol possesses neuroprotective effects with antioxidant mechanisms playing a crucial role, we sought to further explore whether propofol could exert neuroprotection by inhibiting ferroptosis. To this end, after TBI, for the intervention, we injected propofol intraperitoneally at a dosage of 30 mg/kg (Figure 2A). The results showed no significant differences in Nissl body abundance, iron deposition, or the expression of FTH1, Gpx4, and 4‐HNE between the propofol‐treated sham group and the saline‐treated sham group. In contrast, both the TBI + saline and TBI + propofol groups exhibited a significant reduction in Nissl body abundance, increased iron deposition, decreased FTH1 and Gpx4 expression, and increased 4‐HNE expression. In contrast to the TBI + saline group, the TBI + propofol group showed a substantial increase in Nissl body abundance, reduced iron deposition, enhanced FTH1 and Gpx4 expression, and decreased 4‐HNE expression (Figure 2B–J). This indicates that propofol significantly reduced TBI‐induced ferroptosis without causing side damage, thereby confirming the effectiveness of its neuroprotective action.

### 3.3 Propofol Reduces Neuronal Apoptosis by Inhibiting Ferroptosis

**FIGURE 3:**
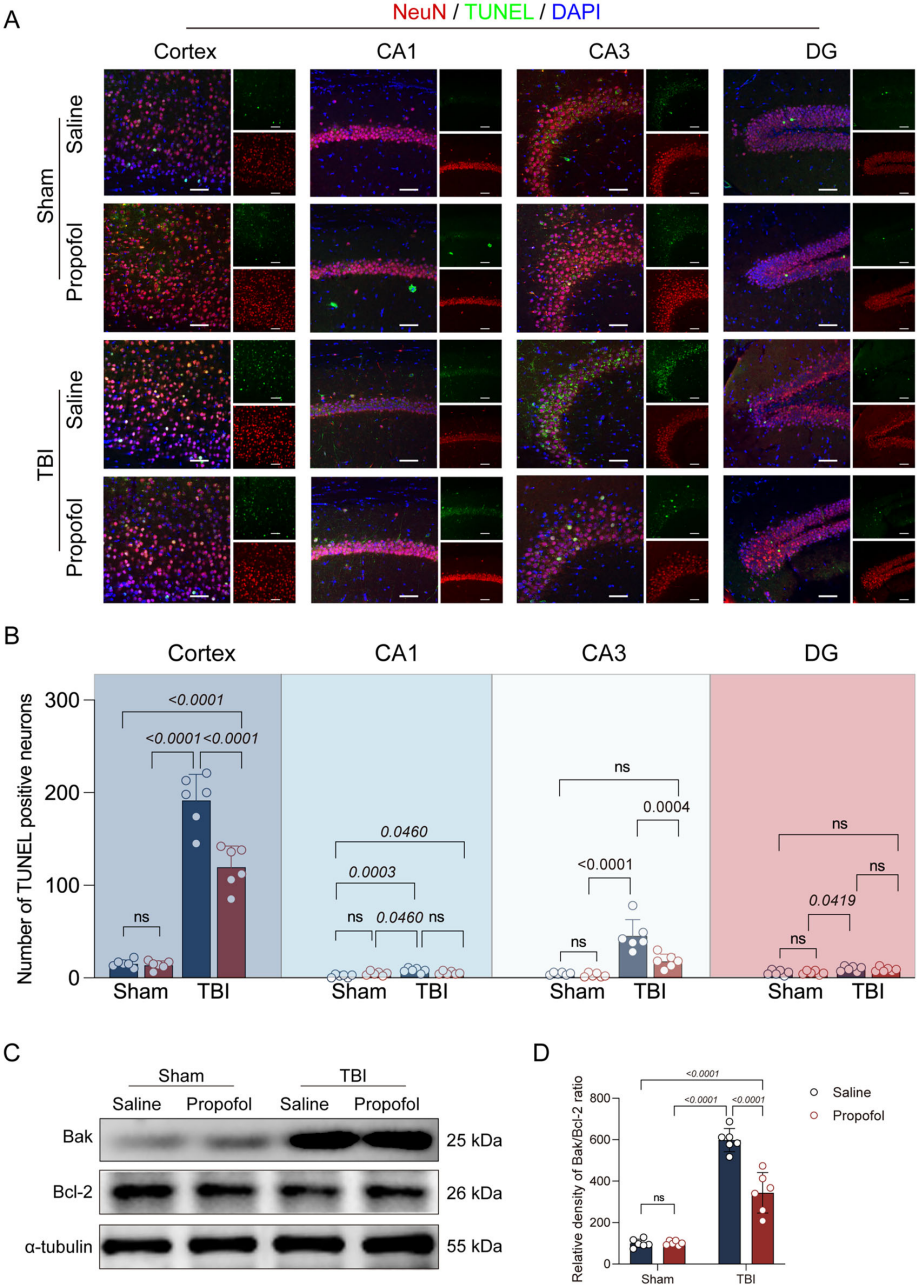
Propofol reduces neuronal apoptosis by inhibiting ferroptosis. (A) Representative TUNEL fluorescence images are displayed for the damaged cortical area, hippocampal CA1 region, CA3 region, and DG region across four groups of mice. In the images, red labeling indicates neurons, green labeling signifies apoptotic cells, and blue labeling represents cell nuclei, with a scale bar set at 50 µm. (B) A comparison of the number of apoptotic neurons in the damaged cortical area, hippocampal CA1 region, CA3 region, and DG region among the four groups of mice is presented (*n* = 6 per group). (C) Representative immunoblots for Bak, Bcl‐2, and α‐tubulin proteins from the four groups of mice are shown. (D) A comparison of the Bak/Bcl‐2 ratio among the four groups of mice is provided (*n* = 6 per group). Data are expressed as the mean ± standard deviation and were statistically analyzed using ANOVA, with *p* values directly indicated on the bar charts.

To further investigate the impact of propofol's inhibition of ferroptosis on neuronal apoptosis, we assessed the apoptotic condition of neurons post‐TBI using TUNEL staining. It was observed that TBI induced substantial neuronal apoptosis in the impacted cortical area and the ipsilateral hippocampal CA3 region, with relatively minor effects on the neurons in the CA1 region and the dentate gyrus (DG) (Figure 3A). The administration of propofol post‐TBI significantly reduced neuronal apoptosis in these areas. Notably, in the sham group, the application of propofol did not increase the incidence of apoptosis (Figure 3A,B). Furthermore, we examined the expression of apoptosis‐related proteins Bcl‐2 and Bak. The results showed no significant difference in the Bak/Bcl‐2 ratio between the sham + saline group and the sham + propofol group. In contrast, the Bak/Bcl‐2 ratio was significantly increased in both the TBI + saline group and the TBI + propofol group. Compared to the TBI + saline group, the TBI + propofol group exhibited a significant decrease in the Bak/Bcl‐2 ratio (Figure 3C,D), indicating that propofol reduced TBI‐induced neuronal apoptosis by modulating apoptosis‐associated proteins.

### 3.4 Propofol Can Inhibit Late‐Onset Neuroinflammation Induced by Ferroptosis

**FIGURE 4:**
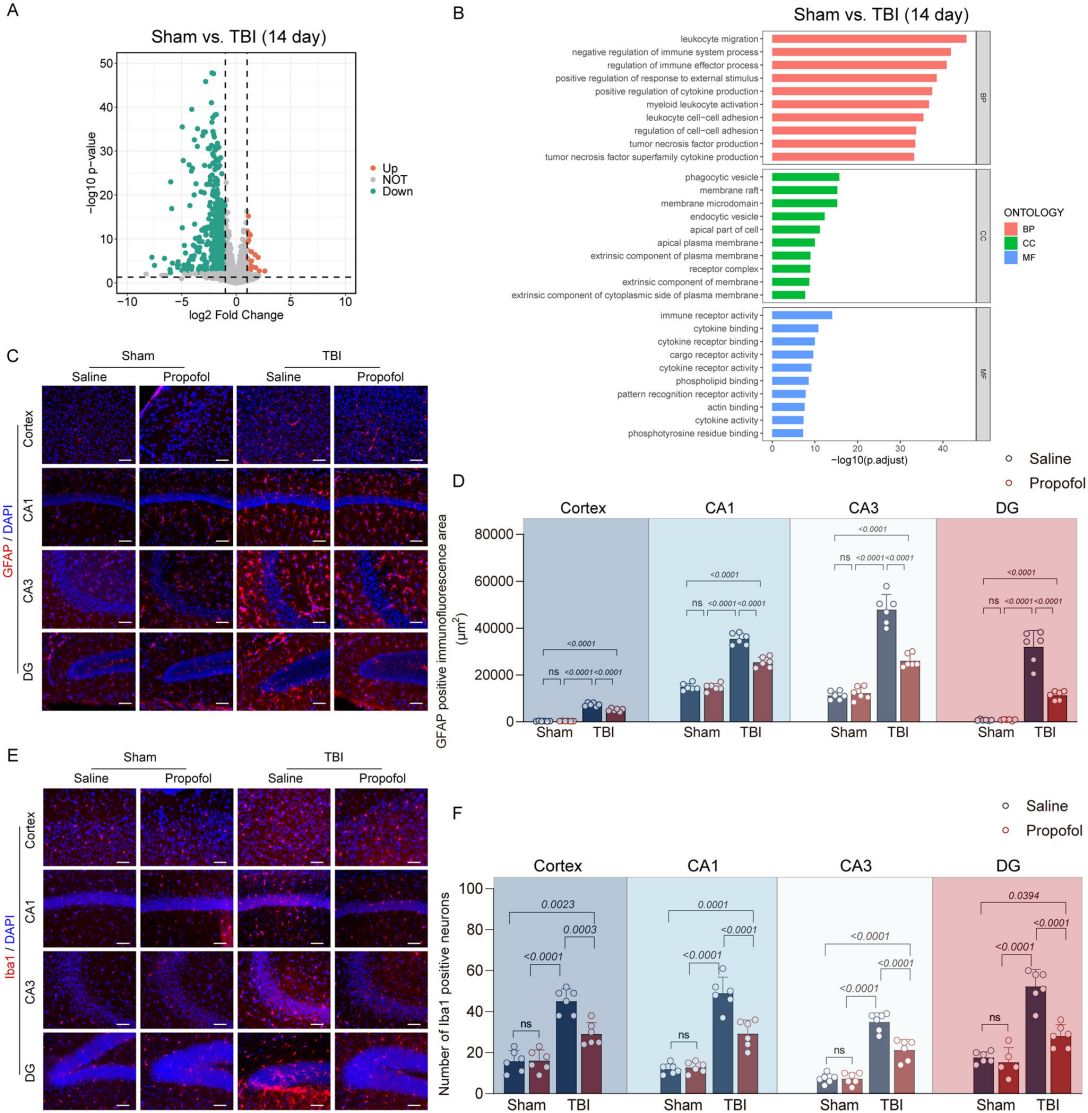
Propofol can inhibit persistent neuroinflammation caused by ferroptosis. (A) At 14 days post‐TBI, a volcano plot illustrates differentially expressed genes between sham and TBI groups, using |log_2_ (fold change, FC)| ≥ 1 and a *p* < 0.05 as thresholds to differentiate upregulated and downregulated genes. (B) Bar charts display the results of Gene Ontology (GO) enrichment analysis. (C) Representative GFAP immunofluorescence images from the damaged cortical area, hippocampal CA1 region, CA3 region, and DG region of mice in the four groups are shown. (D) A comparison of GFAP‐positive area in the damaged cortical area, hippocampal CA1 region, CA3 region, and DG region among the four groups of mice is presented (*n* = 6 per group). E. Representative Iba1 immunofluorescence images from the damaged cortical area, hippocampal CA1 region, CA3 region, and DG region of mice in the four groups are shown. (F) A comparison of the number of Iba1‐positive cells in the damaged cortical area, hippocampal CA1 region, CA3 region, and DG region among the four groups of mice is presented (*n* = 6 per group). Data are expressed as mean ± standard deviation and were statistically analyzed using ANOVA, with *p* values indicated on the bar charts.

The GSE173975 dataset records gene expression variations in the acute (1 day) and later (14 days) phases after TBI. We investigated gene expression variations at 14 days post‐TBI and discovered that 28 genes were upregulated while 526 genes were downregulated (Figure 4A). The DEG modules in later stages were mostly linked to immunological responses, according to GO enrichment analysis, whereas the genes linked to early death recovered to normal levels (Figure 4B). The role of ferroptosis in inflammation is gradually being recognized, and it is more immunogenic than apoptosis. Studies have shown that ferritin deposition in brain injury is considered a key link in the cascade of TBI. This study reveals that immune‐inflammatory responses permeate the entire process of TBI through bioinformatics analysis. Therefore, in the chronic phase of TBI, we examined the changes in astrocytes and microglia, which are sensitive to inflammatory responses. The results indicated that the reactive astrogliosis induced by TBI was more pronounced in the hippocampal CA1, CA3, and DG regions, with less significant changes in the cortical area, possibly related to the distribution and heterogeneity of astrocytes (Figure 4C). Measuring the area of GFAP positivity, we found no significant difference in the cortical area, hippocampal CA1, CA3, and DG regions between the sham + saline group and the sham + propofol group; however, both the TBI + saline group and TBI + propofol group showed a significant increase in GFAP‐positive area. Compared to the TBI + saline group, the TBI + propofol group exhibited a significant reduction in GFAP‐positive fluorescence area (Figure 4D). In addition, using fluorescent area analysis, we assessed the impact of TBI on microglia numbers and found no significant difference between the sham + saline group and the sham + propofol group in the cortical area, hippocampal CA1, CA3, and DG regions; however, both the TBI + saline and TBI + propofol groups had a large increase in microglia numbers. Compared to the TBI + saline group, the TBI + propofol group had a significant reduction in microglia count (Figure 4E,F). This suggests that propofol can suppress the late neuroinflammatory response triggered by TBI.

### 3.5 Propofol Can Improve TBI‐Induced Late‐Onset Cognitive Dysfunction

**FIGURE 5:**
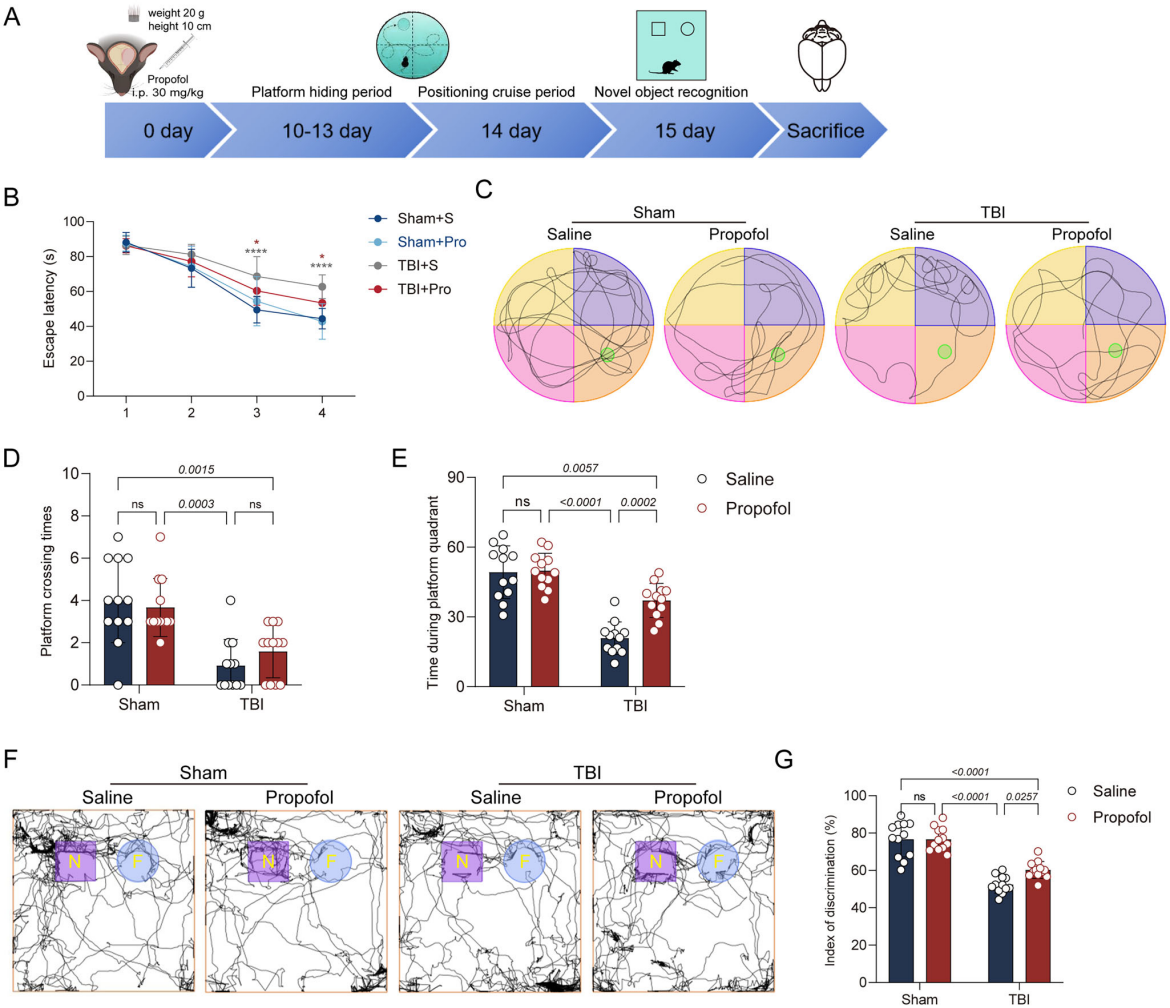
Propofol can improve the long‐term cognitive dysfunction induced by TBI. (A) Schematic of the experimental procedure. After establishing the TBI model, intervention was conducted via intraperitoneal propofol injection (dose: 30 mg/kg). MWM training was conducted from Days 10 to 13 post‐TBI, with testing on Day 14, NOR testing on Day 15, and subsequent brain tissue sampling. (B) Comparison of escape latencies during the MWM training period among the four groups of mice. Statistical analysis was performed using two‐way repeated measures ANOVA, with *p < 0.05 and ****p < 0.0001. (C) Representative traces of the mice's paths in the MWM are shown for the four groups. (D, E) Comparison of platform crossing times and time spent in the target quadrant among the four groups of mice (*n* = 6 per group). (F) Representative traces of the mice in the NOR test are shown for the four groups, with N representing the novel object and F representing the familiar object. (G) Comparison of the discrimination index among the four groups of mice (*n* = 6 per group). Data are presented as mean ± standard deviation and were statistically analyzed using ANOVA, with *p* values indicated on the bar charts.

To assess the impact of early propofol administration on long‐term outcomes by inhibiting ferroptosis, we evaluated the memory functions of mice in various groups during the late phase post‐TBI (10–15 days) using the MWM and NOR tests (Figure 5A). The results showed that propofol significantly improved cognitive dysfunction induced by TBI. During the hidden platform phase of the MWM, the escape latency of mice in the TBI + propofol group was significantly lower than that in the TBI + saline group (Figure 5B). In the probe trial phase, although the number of platform crossings by the TBI + propofol group did not significantly exceed that of the TBI + saline group, the time spent in the target quadrant was significantly increased (Figure 5C–E). To further confirm the memory‐enhancing effect of propofol on TBI mice in the late phase, we conducted the NOR test 1 day after the MWM test. The results showed a significant increase in the discrimination index of mice in the TBI + propofol group compared to the TBI + saline group (Figure 5F,G), indicating the cognitive improvement effects of propofol post‐TBI.

### 3.6 The Neuroprotective Effect of Propofol May Be Related to the Promotion of eNOS‐Derived NO Production

**FIGURE 6:**
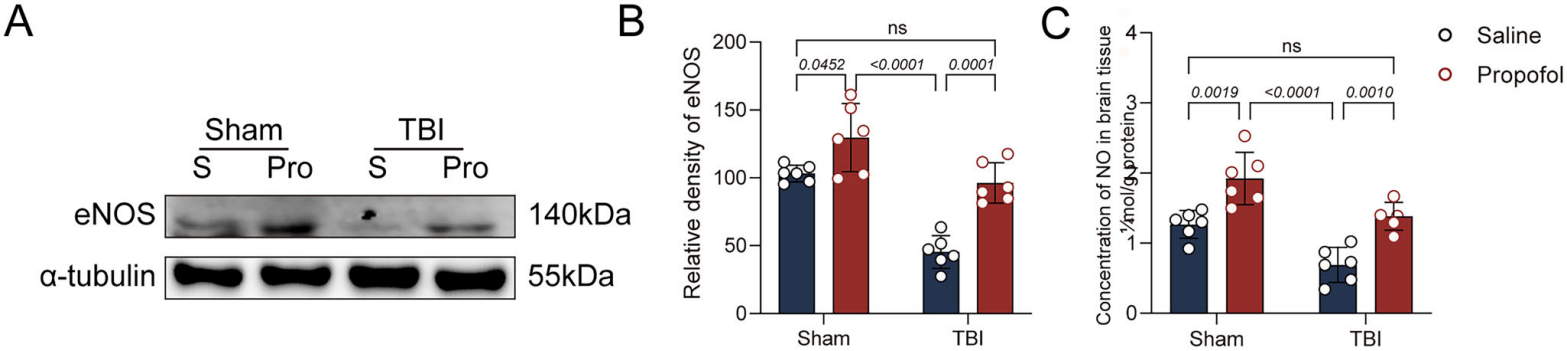
The neuroprotective effect of propofol may be associated with promoting eNOS to produce nitric oxide (NO). (A) Representative immunoblots of eNOS and α‐tubulin proteins from four groups of mice. (B) Comparative analysis of eNOS protein expression among the four groups of mice (*n* = 6). (C) Comparison of NO concentrations in brain tissue among the four groups of mice (*n* = 6). Data are presented as mean ± standard deviation and were statistically analyzed using ANOVA, with *p* values indicated on the bar charts.

Studies have revealed that propofol may exhibit anti‐inflammatory and antioxidant effects by boosting the expression of eNOS, thereby raising the generation of NO (Uskur et al. 2021). Based on this finding, we hypothesized that the significant neuroprotective effect observed for propofol in this study might be related to this mechanism. To test this hypothesis, we measured the expression levels of eNOS protein after propofol administration. The results showed a significant increase in eNOS protein expression in the sham + propofol group compared to the sham + saline group. In contrast, eNOS protein expression was reduced in both the TBI + saline group and the TBI + propofol group, but it was significantly elevated in the latter compared to the former (Figure 6A,B). Furthermore, we determined the concentration of NO in brain tissue and found that the changes in NO concentration were consistent with the expression trends of the eNOS protein. In comparison to the sham + saline group, the NO concentration rose in the sham + propofol group, while it dropped in both the TBI + saline and TBI + propofol groups. The TBI + propofol group had a considerably greater NO concentration than the TBI + saline group (Figure 6C), further supporting the hypothesis that propofol exerts its neuroprotective effect by enhancing eNOS activity.

### 3.7 The eNOS Protein Inhibitor L‐NAME Can Reverse the Early Neuroprotective Effect of Propofol on TBI

**FIGURE 7:**
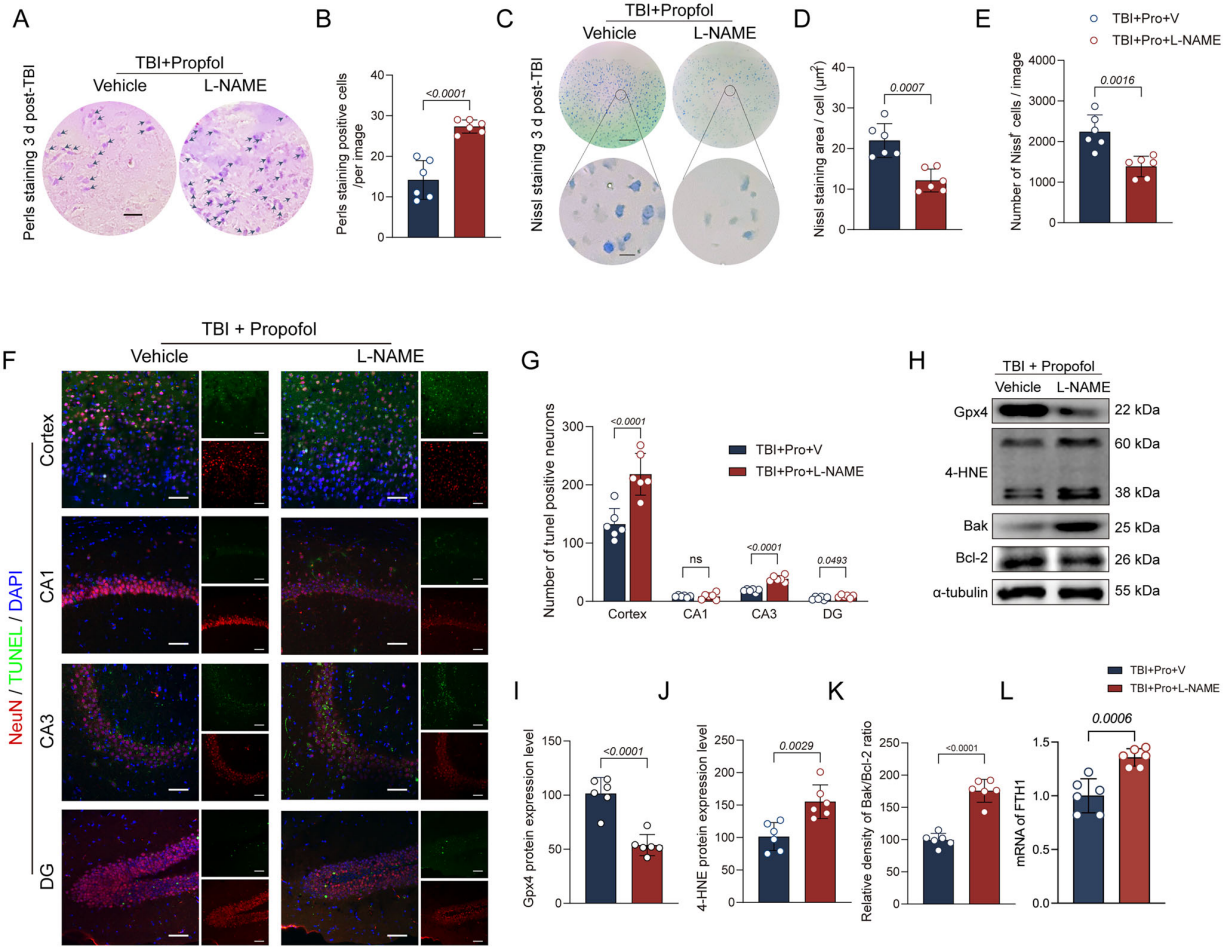
The eNOS protein inhibitor L‐NAME can reverse the early neuroprotective effects of propofol on TBI. (A) Representative images of Perls staining from two groups of mice are displayed. (B) A comparison of the number of positively stained cells in Perls staining between the two groups of mice (*n* = 6 per group) is presented. (C) Representative images of Nissl staining from two groups of mice are shown, with scale bars at 50 or 10 µm. (D, E) Bar graphs illustrate the statistical analysis of Nissl body count and positive area per field of view for four groups of mice (*n* = 6 per group). (F) Representative TUNEL fluorescence images from the damaged cortical area, hippocampal CA1 region, CA3 region, and DG region of two groups of mice are displayed. Red labeling indicates neurons, green labeling indicates apoptotic cells, and blue labeling indicates cell nuclei, with a scale bar at 50 µm. (G) A comparison of the number of apoptotic neurons in the damaged cortical area, hippocampal CA1 region, CA3 region, and DG region among four groups of mice (*n* = 6 per group) is presented. (H) Representative immunoblots of Gpx4, 4‐HNE, Bak, Bcl‐2, and α‐tubulin proteins from two groups of mice are shown. (I–K) Comparisons of Gpx4 protein expression, 4‐HNE protein expression, and the Bak/Bcl‐2 ratio between the two groups of mice (*n* = 6 per group) are provided. (L) Comparison of the expression levels of FTH1 mRNA between the two groups of mice (*n* = 6 per group). Data are presented as mean ± standard deviation and statistically analyzed using Student's *t*‐test, with *p* values on the bar charts.

To further investigate whether propofol inhibits ferroptosis by promoting eNOS expression, we utilized the eNOS inhibitor L‐NAME in our experiments. The study found that administering L‐NAME dramatically negated the early neuroprotective effects of propofol on TBI. Specifically, compared to the group treated with propofol only after TBI (TBI + propofol + V), the group treated with L‐NAME (TBI + propofol + L‐NAME) exhibited a significant increase in iron deposition, a marked decrease in Nissl body density, and a significant increase in neuronal apoptosis in the cortical area and the hippocampal CA3 region. Furthermore, the expression of FTH1 and Gpx4 protein was reduced, the expression of 4‐HNE proteins was elevated, and the Bak/Bcl‐2 ratio was also decreased. These findings suggest that propofol may exert its neuroprotective effects by activating eNOS (Figure 7A–L).

### 3.8 The eNOS Protein Inhibitor L‐NAME Can Reverse the Propofol‐Mediated Improvement in Long‐Term TBI Prognosis

**FIGURE 8:**
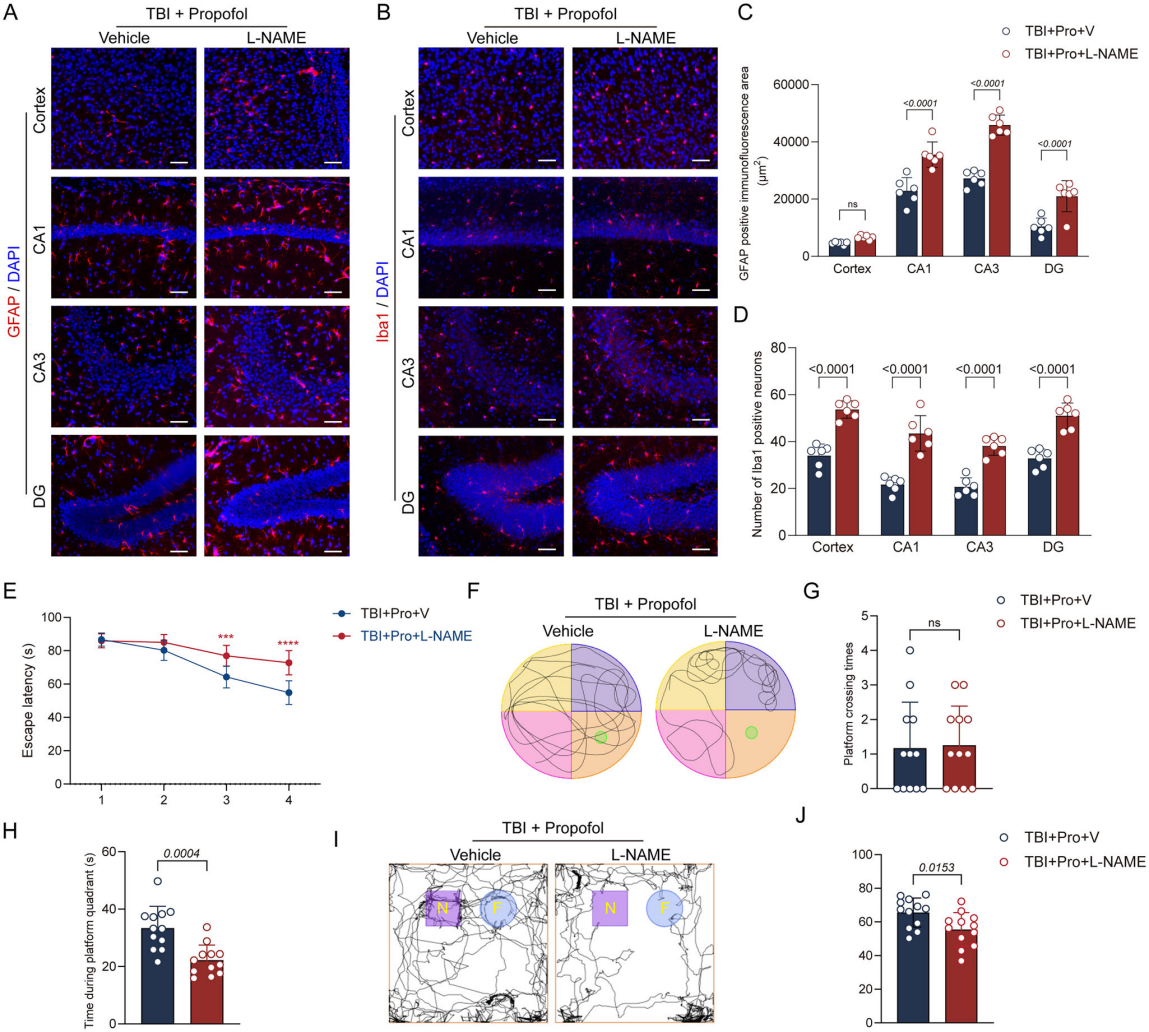
The eNOS protein inhibitor L‐NAME can reverse the effect of propofol in improving long‐term outcomes after TBI. (A, B) Representative GFAP and Iba1 immunofluorescence images from the damaged cortical area, hippocampal CA1 region, CA3 region, and DG region of two groups of mice are shown. (C, D) Comparisons of GFAP‐positive area and Iba1‐positive cell counts in the damaged cortical area, hippocampal CA1 region, CA3 region, and DG region between two groups of mice are presented (*n* = 6 per group). (E) The escape latencies during the MWM training period for two groups of mice were compared, and two‐way repeated measures ANOVA was used for statistical analysis, with ****p < 0.0001. (F) Representative traces of the mice in the MWM are shown. (G, H) Comparisons of platform crossing times and time spent in the target quadrant between two groups of mice are presented (*n* = 6 per group). (I) Representative traces of the mice in the NOR test are shown, with N representing the novel object and F representing the familiar object. (J) A comparison of the discrimination index between two groups of mice is presented (*n* = 6 per group). Data are expressed as mean ± standard deviation and were statistically analyzed using Student's *t*‐test, with *p* values indicated on the bar charts.

To further investigate the impact of the L‐NAME inhibitor on long‐term TBI prognosis, we conducted immunofluorescence experiments. The results showed a significant increase in GFAP‐positive area in the hippocampal CA1 region, CA3 region, and DG region of the group treated with L‐NAME in addition to propofol (TBI + propofol + L‐NAME) compared to the group treated with propofol only after TBI (TBI + propofol + V), indicating enhanced reactive astrogliosis. In addition, the number of microglia across the brain increased significantly (Figure 8A–D). Behaviorally, compared to the TBI + propofol + V group, the TBI + propofol + L‐NAME group exhibited increased escape latency, reduced time spent in the target quadrant, and a decreased discrimination index in the NOR test (Figure 8E–J), suggesting that the administration of L‐NAME negates the ameliorative effects of propofol on the long‐term prognosis of TBI.

## 4 Discussion

TBI is a common neurosurgical emergency, with its high mortality and disability rates imposing a heavy burden on families and society (Haarbauer‐Krupa et al. 2021; Schneider et al. 2023). The pathophysiological mechanisms of TBI are highly complex, involving both primary and secondary injuries. Primary injury refers to the irreversible mechanical damage to brain tissue and surrounding structures caused by the initial impact (Robinson 2021). Subsequently, secondary brain injury develops, characterized by further pathological and physiological changes that damage brain tissue (Hu et al. 2022; H. Wu et al. 2021), including mechanisms such as inflammation, oxidative stress, apoptosis, mitochondrial dysfunction, and calcium overload (Lai et al. 2022; Zhang et al. 2023). Notably, secondary injury has the potential for reversibility (Witcher et al. 2021), making early intervention and appropriate treatment strategies crucial for achieving the maximum repair of reversible damage (H. T. Miao et al. 2023; Qin et al. 2022).

Emerging research has identified “ferroptosis” as a form of programmed cell death distinct from apoptosis, observed in various diseases including stroke, postoperative cognitive dysfunction, Alzheimer's disease, and Parkinson's disease (Bao et al. 2021; J. Wu et al. 2020; Xie et al. 2019). In this study, through bioinformatics analysis, we found abnormal expression of genes related to neuronal death and iron transport in the acute stage of TBI, suggesting that ferroptosis may be a significant contributor to secondary brain injury in TBI. Therefore, we induced TBI by impacting the exposed dura mater with a falling weight and detected iron ion deposition as well as the expression of Gpx4 and 4‐hydroxynonenal (4‐HNE) proteins at 3 days post‐TBI—coinciding with the peak period of ferroptosis. The results showed significant ferroptosis events following TBI, consistent with the research by Q. Li et al. (2017). Recent studies have established 4‐HNE as a key molecule in ferroptosis associated with oxidative stress and inflammatory diseases (Baechler, Bloemberg, and Quadrilatero 2019; Y. Li, Liu, and Qi 2018). Gpx4 is an antioxidant enzyme belonging to the glutathione peroxidase family, maintaining intracellular REDOX balance by eliminating oxidative stress factors produced by lipid peroxidation (Jia et al. 2020). However, in conditions related to ferroptosis, the balance between 4‐HNE and Gpx4 may be disrupted (P. Liu et al. 2020), with reduced Gpx4 function potentially making cells more susceptible to oxidative damage due to hindered removal of oxidative stress. Excessive accumulation of 4‐HNE may trigger inflammatory responses and exacerbate cell damage. Our study observed decreased Gpx4 expression and increased 4‐HNE expression in the brain tissue of TBI‐exposed mice, echoing these findings.

Propofol is a short‐acting intravenous anesthetic of the alkylphenol class, primarily used in clinical practice for total intravenous anesthesia (Zhu et al. 2023). It is renowned for its rapid onset, high potency, and excellent controllability. Numerous studies have demonstrated that propofol possesses neuroprotective properties. In our study, after the administration of propofol, we assessed indicators of ferroptosis and found that propofol significantly reduced iron deposition and the occurrence of ferroptosis. Furthermore, by using Nissl staining to evaluate neuronal function, we discovered that propofol markedly improved the reduction in Nissl body density induced by TBI. We also observed neuronal apoptosis using TUNEL staining. A significant increase in neuronal apoptosis in the impacted cortical area was expected. More intriguingly, we found a significant increase in neuronal apoptosis in the hippocampal CA3 region of mice that had experienced TBI. This may be related to the CA3 region being rich in mossy cells, which receive substantial glutamatergic input from granule cells and are thus susceptible to excitotoxic damage (Ratzliff et al. 2002). Another possibility is that the main force of TBI was concentrated in the CA3 area, leading to higher pressure in this region and consequently significant neuronal apoptosis. Accordingly, the neuroprotective effects of propofol were more pronounced in the cortex and hippocampal CA3 area. In the sham group, the application of propofol did not lead to an increase in neuronal apoptosis, differing from the results of L. Sun's study (Liang et al. 2022; L. Sun et al. 2019). This discrepancy might be due to the lower dose of propofol we used, which did not induce neurotoxicity, or it could be that the concurrent use of sevoflurane masked the potential neurotoxic effects of propofol in normal tissue.

Recent studies have indicated that ferritin deposition in cranial brain injuries is considered a key link in the TBI cascade (J. Li et al. 2022.). Therefore, the early inhibition of ferroptosis may offer significant benefits for long‐term prognosis. Consequently, we focused on the changes in long‐term outcomes in TBI mice following the acute‐phase administration of propofol. Bioinformatics analysis has revealed that immune‐inflammatory responses persist throughout the entire process of TBI. The role of ferroptosis in inflammation is gradually being recognized. It is more immunogenic than apoptosis, and studies have indicated that ferritin deposition in brain injury is considered a key link in the TBI cascade reaction, which is related to the complex interaction between ferroptosis and inflammation. Studies have shown that ROS and lipid peroxidation products (LPO) generated during ferroptosis can activate inflammatory responses, leading to the release of inflammatory factors, such as TNF‐α, IL‐1β, and IL‐6. Other studies have indicated that the rupture of ferroptotic cells can release damage‐associated molecular patterns (DAMPs), which can activate the NF‐κB pathway by binding to receptors such as the receptor for advanced glycation end products (RAGE), thereby promoting inflammatory responses. Inflammation, in turn, can act on ferroptosis. The activation of inflammation‐related signaling pathways, such as JAK‐STAT, NF‐κB, inflammasomes, cGAS‐STING, and MAPK signaling pathways, is closely related to the occurrence of ferroptosis, and the activation of these signaling pathways can promote ferroptosis. Ferroptosis, in turn, can further activate these signaling pathways by releasing DAMPs, forming a vicious cycle (J. Chen, Shi, et al. 2024; Deng et al. 2023). This study reveals through bioinformatics analysis that immune‐inflammatory responses permeate the entire process of TBI. Therefore, in the chronic phase of TBI, we examined the changes in astrocytes and microglia, which are sensitive to inflammatory responses. Thus, in the chronic phase of TBI, we examined the changes in astrocytes and microglia, which are sensitive to inflammatory responses. Most studies have shown that astrocytes undergo reactive gliosis with significant morphological changes in response to inflammatory stress (X. L. Liu et al. 2023). We used immunofluorescence to reflect changes in astrocytes and found significant alterations in the hippocampal CA1, CA3, and DG regions, with less change in the cortical area. The cortical GFAP‐positive areas were primarily composed of end‐feet constituting the blood‐brain barrier, and this situation may be related to the special distribution and heterogeneity of astrocytes. Due to the complex and variable expression profile of microglia, differentiating microglial phenotypes in this study posed certain difficulties. Therefore, we chose to preliminarily explore the changes in microglia caused by TBI by observing the changes in the number of microglia (Y. Chen et al. 2021; Sousa et al. 2018). The results showed that TBI can cause the proliferation of microglia throughout the brain, and propofol can significantly reduce the number of microglia. Persistent inflammatory states are considered an important pathogenic mechanism for brain function decline (Gulen et al. 2023). To reflect brain function, we assessed the memory of mice through behavioral testing; the results from the water maze indicated that mice experiencing TBI had extended escape latencies, reduced platform crossings, and decreased time spent in the target quadrant, consistent with the findings of X. Qu et al. (2021). After propofol administration, the escape latency of mice was significantly reduced. Although there was no statistical difference in platform crossings, the time spent in the target quadrant significantly increased, suggesting that time spent in the target quadrant might be a more sensitive measure for assessing memory function than platform crossings. To further clarify the impact of propofol's inhibition of ferroptosis on long‐term memory function, we conducted a NOR test. The results showed that propofol application significantly improved the decline in the discrimination index caused by TBI. These findings suggest that propofol significantly improves the long‐term prognosis of TBI by inhibiting ferroptosis.

We have developed a keen interest in the mechanism by which propofol inhibits ferroptosis. Numerous studies have suggested that propofol can dose‐dependently enhance the expression of eNOS, therefore boosting NO production (L. Wang et al. 2010). As a ubiquitous gas molecule in the central nervous system, NO has been shown to react with oxygen free radicals to form more stable compounds, reducing free radical activity and alleviating oxidative stress (Radi 2018). Moreover, NO can prevent lipid peroxidation, thus protecting the integrity of the cell membrane. Consequently, we further examined the effects of propofol on eNOS protein expression and NO levels, confirming that propofol treatment significantly increased both eNOS protein expression and NO content. Recent research by Kapralov et al. has also found that NO donors can enhance cellular resistance to RSL3‐induced ferroptosis (Mikulska‐Ruminska et al. 2021). To clarify whether propofol inhibits ferroptosis through the eNOS/NO signaling pathway, we administered the eNOS inhibitor L‐NAME intravenously before propofol injection via the abdominal cavity. The results demonstrated that L‐NAME abolished propofol's neuroprotective effects.

Although our study innovatively explored the impact of propofol on the early and long‐term prognosis of TBI, there are still some limitations. For instance, we did not dynamically monitor the changes in NO concentration, a problem that may be resolved with the development of future NO probe technology. In addition, this experiment only measured changes in the number of microglia, without an in‐depth study of changes in their expression profiles. We plan to continue in‐depth exploration in this field in the future.

## 5 Conclusion

In summary, ferroptosis is a critical component of the TBI cascade. Propofol modulates the expression of eNOS, thereby promoting the production of NO, inhibiting ferroptosis, and significantly improving the prognosis of TBI.

## Author Contributions

**Zi‐Lei Zheng**: writing–original draft, formal analysis. **Xu‐Peng Wang**: writing–original draft, formal analysis. **Yu‐Fei Hu**: methodology, project administration. **Wen‐Guang Li**: investigation, data curation. **Qi Zhou**: data curation, investigation. **Fang Xu**: investigation, data curation. **Qiu‐Jun Wang**: funding acquisition, visualization, writing–review and editing.

## Ethics Statement

All experiments were approved by the Ethics Committee of the Third Hospital of Hebei Medical University under the registration number Z2022‐034‐1.

## Conflicts of Interest

The authors declare no conflicts of interest.

### Peer Review

The peer review history for this article is available at https://publons.com/publon/10.1002/brb3.70187

## Supporting information



Supplementary Materials.

## Data Availability

All data included are available reasonable demand to the corresponding authors in this study.
