# Supplementary material for: Propofol Suppresses Ferroptosis via Modulating eNOS/NO Signaling Pathway to Improve Traumatic Brain Injury
Source: Brain Behav. 2024 Dec 26;14(12):e70187. doi: 10.1002/brb3.70187 (PMC11671348; doi:10.1002/brb3.70187)
Supplement: Supplementary file 1 — Supplementary Materials. [file BRB3-14-e70187-s001.docx]

| FTH1 | Forward primer | CAAGTGCGCCAGAACTACCA |
| --- | --- | --- |
|  | Reverse Primer | TCAGAGCCACATCATCTCGG |
| GAPDH | Forward primer | CCCTTAAGAGGGATGCTGCC |
|  | Reverse Primer | TACGGCCAAATCCGTTCACA |

Table 1. Primer Information
